# The Effects of Perfluorooctanesulfonic acid (PFOS) on Human Umbilical Vein Endothelial Cells (HUVECs) Proliferation and Gene Expression and its Implications on Fetal Development

**DOI:** 10.17912/micropub.biology.001318

**Published:** 2024-12-03

**Authors:** Alycia Ashby, Patrick Murphy, James Jukosky, Chery A. Whipple

**Affiliations:** 1 Colby–Sawyer College, New London, New Hampshire, United States

## Abstract

Polyfluoro-alkyl substances (PFAS) are widely distributed environmental contaminants linked to human toxicity and developmental delays, especially low birthweight (LBW). In this study, Human Umbilical Vein Endothelial Cells (HUVECs) were exposed to the PFAS perfluorooctanesulfonic acid (PFOS). After 48-hours, their proliferation, and differential gene expression were assessed. A small, yet significant, reduction in proliferation was seen at 50 μg/mL and 75 μg/mL. RNA sequencing showed that estrogen response and notch signaling pathways were significantly altered. This study increases our understanding of how PFAS may interfere with endothelial cell (HUVECs) functions which may have larger effects on fetal growth, development, and birthweight.

**Figure 1. Effect of HUVEC Exposure to PFOS on Cell Proliferation and Gene Expression:**
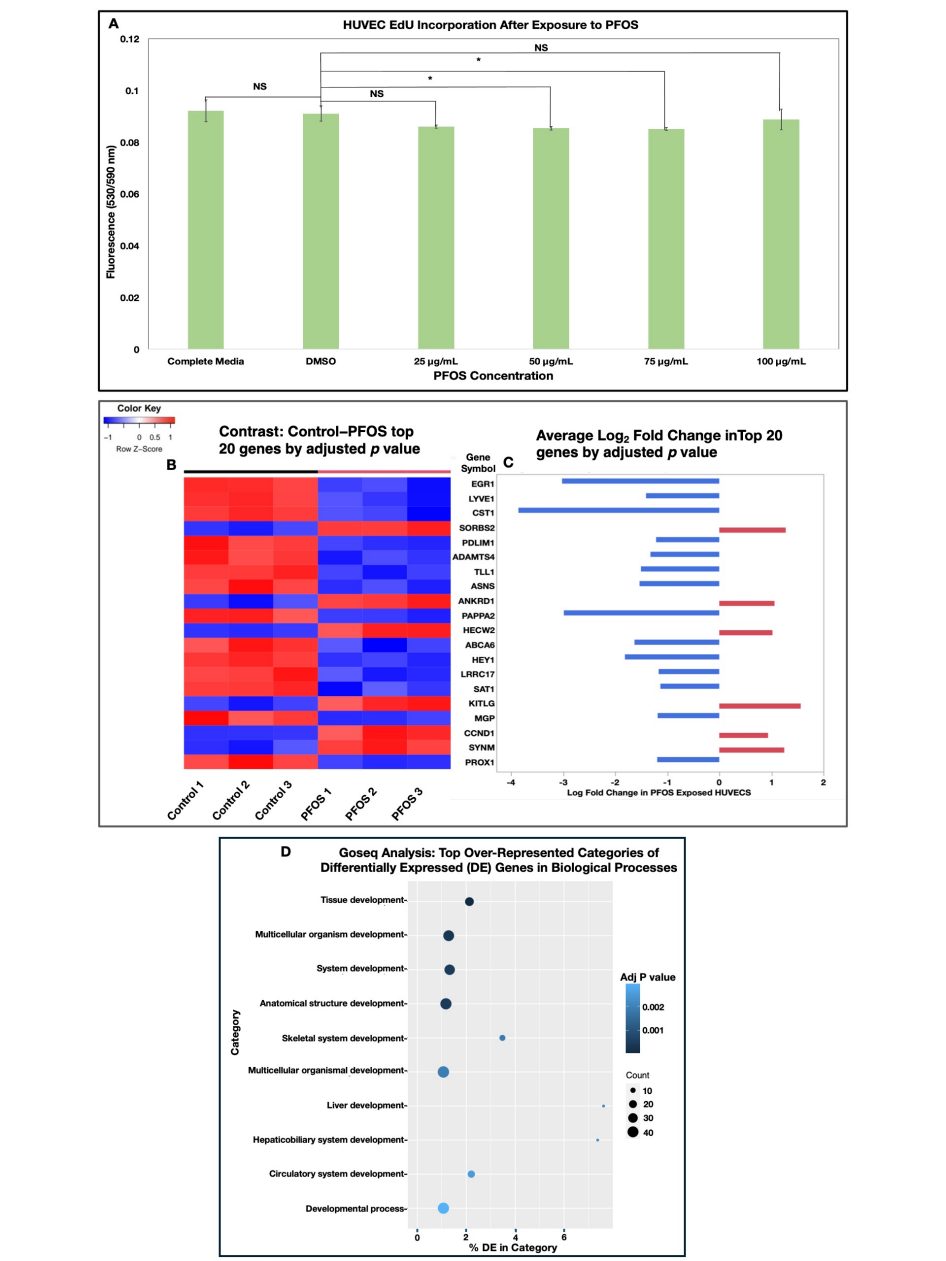
(A) HUVECs were treated with PFOS treatments (Complete media, 0.6% DMSO, 25 μg/mL, 50 μg/mL, 75 μg/mL, and 100 μg/mL PFOS) along with the EdU thymine analog for 48 hours. Post treatment the Click-it EdU protocol was followed to measure the amount of fluorescence from the incorporated EdU thymine into the cell’s DNA. The Click-it Edu experiment was conducted three independent times with five wells for each treatment group. In an ANOVA (F _5, 84_ = 3.92, *p* = 0.003) followed by a post-hoc Tukey test (α = 0.05), PFOS had significant reduction in proliferation in the 50 μg/mL and 75 μg/mL compared to the control (complete media and DMSO). NS = not significant, * = *p* < 0.05, n=3. (B) Heatmap of the top 20 differentially expressed genes by adjusted *p* value between media control and 50 μg/mL PFOS after 48 hours of exposure. (C) Average log _2_ fold change in the top 20 differentially expressed genes (by adjusted *p* value) in PFOS exposed HUVECs compared to control HUVECs. Differential expression was compared using limma-voom, and lowly expressed genes were removed. Contrasts measured in limma-voom were Control versus PFOS. Contrast testing was set to a minimum log _2_ fold change of 0.58 and a *p* -value adjusted threshold of 0.01, significance was tested relative to a fold-change threshold. (D) GOseq pathway analysis displaying the top ten over-represented categories of differentially expressed genes in biological processes in HUVECs after 50 μg/mL of PFOS for 48 hours

## Description

Perfluoroalkyl and polyfluoro-alkyl substances, also widely known as PFAS, are a class of chemicals that are distributed worldwide. PFAS have been produced by manufacturing companies to create various products such as water-resistant coatings for clothes, furniture, and cookware, as well as being used in firefighting foam (Jian et al., 2018; Sunderland et al., 2018; Bonato et al, 2020) . PFAS have a long half-life contributing to their persistence in the environment and hazardous impact on humans. Human exposure has been linked to cardiovascular disease, thyroid dysfunction, immune dysfunction, and cancer (Fenton et al, 2020; Maddalon et al., 2023) . PFAS have been seen to disrupt a wide range of cellular processes including alterations in oxidative stress, cellular metabolism, and prenatal development (Wielsøe et al, 2015; Bonato et al., 2020; Marin et al., 2024). A highly prevalent PFAS that humans are primarily exposed to is Perfluorooctanesulfonic acid (PFOS) (Kang et al., 2021) . PFOS, is characterized with a carbon fluorinated chain ending with a sulfonic acid group. In areas of high exposure, PFAS have been linked to the development of low birthweight (LBW) with studies showing an inverse relationship between PFOS exposure and LBW (Chen et al., 2012; Gundacker et al., 2022) . LBW is a concern because it puts the baby at high risk for complications such as infection, breathing problems, digestive and nervous system abnormalities, and may lead to sudden infant death (Osuchukwu and Reed, 2024) . Little research has been done to understand the mechanistic cause of these adverse birth defects in response to PFOS. Research has delineated potential causes, but supporting data are limited. According to one study the maternal serum concentration range of PFOS was 1.04–16.66 ng/ml, while the ranges for the placenta and various fetal tissue such as the liver, lung, heart, central nervous system, and adipose tissue was 0.45–3.87 ng/g and 0.19–12.61 ng/g respectively (Mamsen et al., 2019) . However, environmental concentrations of PFOS exposure range vastly depending on the area. For example, a region in Veneto Italy found that people within the area had ranging concentration between 2.72 and 70.27 ng/g PFOS (Ingelido et al., 2018) . Specific occupations and related work areas such as military sites, airports, wastewater treatment plants, and industrial facilities are prone to high exposure (Salvatore et al., 2018) ; PFOS in particular has been found in aquatic ecosystems in high population areas in the U.S. and Europe at a range of 97 ng/L to 1371 ng/L (Loos et al., 2009; Jarvis et al., 2021; and Zhang et al., 2016) . At high contamination sites, such as U.S. Air Force bases, soil concentrations have been detected at levels as high as 460,000 µg/kg (Brusseau et al. 2020) . These values are important given that PFOS is a forever chemical that bioaccumulates in the environment and in the body. While a biologically relevant dose is difficult to determine, it is known that interference of blood vessel development from the placenta to the developing embryo due to PFOS exposure could lead to less oxygen and nutrients being transferred thus potentiating LBW. In this study we wanted to understand how PFOS may be interfering with vascularization. One important tissue for supporting the developing fetus is the umbilical cord (Burton et al., 2015) . To examine how PFOS is affecting this vasculature, Human Umbilical Vein Endothelial Cells (HUVECs) were used as the model system. We examined how endothelial cells proliferation and differential gene expression (RNA-SEQ) were affected after exposure to PFOS. Our working concentrations of PFOS ranged from 25 ug/mL – 100 ug/mL which compares well with concentrations used in other *in-vitro* experiments (Midgett et al. 2014; Sonkar et al. 2019; and Zarei et al. 2018) .

PFOS exposure showed a significant reduction in HUVEC proliferation ( Figure 1A ). Additionally, the experiment showed that the inhibition of proliferation leveled off as the concentration of PFAS increased. From this assay we observed a significant decrease in proliferation at 50 μg/mL and 75 μg/mL PFOS. Although we did not observe a significant decrease in proliferation at the highest concentration of PFOS; a similar study showed a dramatic effect on endothelial cell after PFOS exposure reporting a 60% reduction in cell counted when compared to their control (0.5% EtOH). However, this study exposed the endothelial cells to 72 μg/mL for 7 days instead of the acute 48 hours of exposure our HUVECs experienced. Additionally, their study utilized a 3D co-culture of HUVECs and fibroblasts to examine how PFOS affects angiogenic sprouting as seen by the number of tips and branches created. They saw a negative relationship with an increase concentration of PFOS reducing outgrowth (Forsthuber et al., 2022) .

Given that the lowest concentration we observed significant changes in proliferation was at 50 μg/mL PFOS, this concentration was used for a 48-hour exposure, followed by extraction of RNA from control and exposed HUVECs, and RNA sequencing of the RNA isolated from the HUVECs. As seen in Figure 1 (B and C), 50 μg/mL PFOS had a significant effect altering the gene expression of HUVECs after 48 hours of exposure compared to control cells. This acute exposure demonstrated a largely down regulatory effect on gene expression. Analysis showed that there was a downregulation in EGR-1, HEY1, and SAT1 these are genes involved in cell proliferation and angiogenesis. However, some genes that promote proliferation were upregulated including CCND1, KITLG, and HECW2. Additionally, PFOS exposure can be seen to alter gene expression related to the cytoskeleton (SORB2, SYNM, PDLIM1), lymphatic development (LYVE1, PROX1), metabolism (ASNS, SAT1, PAPPA2), extracellular matrix (ADAMTS4, LRRC17, TLL1, MGP, CST1), cardiovascular development (HEY1, ANKRD1, TLL1) and lipid transport (ABCA6).

A GOseq analysis was done to examine what pathways this acute PFOS exposure had on HUVECs (Figure D). According to this analysis the top 10 pathways from exposure are all involved in development. An additional HALLMARK and KEGG pathway analyses were performed to provide insight into pathways that PFOS may be acting through to cause developmental changes. Similar pathways between each analysis were those involved with proliferation such as DNA replication and repair, E2F checkpoint, and G2M check point. These data indicate that PFOS are disrupting pathways relating to cellular proliferation which are similar to other studies delineating that PFOS induces cell cycle arrest at the G0/G1 checkpoint (Qu et al., 2021; Otero-Sabio et al., 2022) . Pathways specific to KEGG indicated interference with metabolism specifically with alanine, aspartate and glutamate metabolism as well glycine, serine, and threonine metabolism. HALLMARK specific pathways suggest PFOS is disrupting estrogen response early, unfolded protein response, and TNFA signaling via NF-κB. Similar studies investigating the cellular effects of PFOS have implicated it in inducing oxidative stress, however our pathway analysis did not indicate a reactive oxygen species pathway or a change in characteristic genes such as Nrf-2, Gpx, Txn, or other antioxidant genes (Qian et al., 2010; Lee et al., 2012; Elumalai et al., 2023) .

This study has shown that exposure of endothelial cells to 50 μg/mL of PFOS elicits a significant change in proliferation. While this change was small, we detected significant modulations of the expression of genes in signaling pathways involved in cell cycle regulation and development. The ability for PFOS to disrupt endothelial cell proliferation may elicit a reduction in vascularization. Reduced vascularization is one indicator of placental insufficiency. Placental insufficiency is linked to many obstetric conditions, such as the reduction in fetal birthweight (Wardinger and Ambati, 2024) . Future studies can further explore the implicated genes and downstream signaling pathways of PFOS from this study. For example, one pathway that may provide more insight into the proliferative and developmental effects of PFOS exposure is the estrogen response pathway indicated by the HALLMARK analysis. PFOS-estrogen interactions are well studied and thus far has had varying results on PFOS effects through estrogen receptors (ER). For example, some studies suggest that PFOS acts as an ERα agonist or has weak affinity to ERα (Du et al., 2012; Gao et al., 2012; Behr et al., 2018) . One study suggests that PFOS does not act on ERα, instead their study suggests that PFOS interacts with ERβ. Additionally, one study stated that PFOS interacts with ERβ, but through ERβ’s non-genomic pathway to reduce proliferation by inhibiting Erk1/2 activation (Xu et al., 2017; Qu et al., 2021) . Estrogen receptors are important mediators of endothelial function, and both alpha and beta variants are present within vascular cells (Chakrabarti et al., 2014) . If ERβ is inhibiting Erk1/2 then it could explain the decreased expression of the EGR-1 transcription factor which is responsible for early-immediate gene expression involved with growth and development (Khachigian, 2021) . Future studies could examine PFOS and ER interaction within HUVECs though western blot to determine the levels of these receptors within the cells as well as through an ER luciferase reporter to examine how PFOS influences their activity.

An additional pathway that PFOS may be interfering with is notch signaling as indicated by the decreased expression of HEY1 (Figure B). HEY1 is a transcription factor that is expressed upon notch activation. Notch is an important pathway for development as well as for angiogenesis (Akil et al., 2021) . Studies have demonstrated that HEY1 expression is critical for embryonic development, especially cardiovascular development (Fischer et al., 2004; Watanabe et al., 2020) . Additionally, notch plays an important role in angiogenesis dictating their role as stalk or tip cells during the process. Interference with this signaling causes a loss of their hierarchical organization inducing a failure for proper tube formation and junctional integrality (Mack and Iruela-Arispe, 2018) . There have been studies demonstrating that other prevalent PFAS have varying degrees of influence on notch. For example, PFOA enhances notch signaling while PFBS, appears to have no interference with notch signaling (Marinello et al., 2020, Poteser et al., 2020) . There are limited studies investigating PFOS’ effect on notch signaling, however, research conducted by Zhao et al indicated that PFOS disrupts notch signaling in early development (2024). This encourages future studies to investigate notch activity and PFOS exposure.

This experiment largely looked at the effects of PFAS on the vascular cells that comprise the placenta. To gain more information on how PFAS may influence the placenta and thus fetal development, placenta cells could be used. The addition of conducting similar experiments on placental cells would provide complementary information alongside the HUVECs providing a bigger picture on the molecular level on how PFAS affects the placenta as a whole organ. It is important to note that we are not exposed only to a single PFAS, instead there is often a mixture of PFAS in human blood serum. In a study conducted in 2007 that examined serum samples from a National Health and Nutrition Examination Survey, the four PFAS that were found among 98% of the samples was PFOS, PFOA, PFHxS, and PFNA (Calafat, et al., 2007) . One study found that a mixture of PFOS and another prominent PFAS, PFOA had synergistic carcinogenic effects on Human Breast epithelial cells (Pierozan et al., 2023) . Building off this experiment future studies could investigate how a combination of PFAS effect HUVECs to determine if there is a similar synergistic effect for LBW development.

## Methods

**Tissue Culture**

HUVECs were incubated at 37C with 5% CO _2_ and maintained in complete media (2% FBS plus growth factors: rh VEGF, rh EGF, rh FGF basic, rh IGF-1, L-glutamine, Heparin sulfate, Hydrocortisone hemisuccinate, Ascorbic acid, as well as penicillin and streptomycin) provided by ATCC (Manassas, Virginia).

**Click-iT™ EdU Cell Proliferation**

HUVECs were plated at a density of 7,000 cells per well in the 96-well plate and exposed to range of PFOS concentrations (25 μg/mL, 50 μg/mL, 75 μg/mL, 100 μg/mL). This range is similar to other in-vitro experiments examining PFOS cellular effects (Midgett et al., 2015; Sonkar et al., 2019; Forsthuber et al., 2022) . The proliferation was assayed in a 96-well plate using three independent Click-it EdU experiments with 5 replicates performed for each replicate. According to the protocol provided by Invitrogen, HUVECs will be exposed to the desired concentration gradient with the thymidine analog added to the experimental media for 48hrs. To activate the EdU fluorescence, HRP-azide was added to each well, this attaches to the thymidine analog. The signal is further amplified by an anti-Oregon Green® antibody conjugated to the horseradish peroxidase (HRP), which then reacts with Amplex® UltraRed and produces a red fluorescent product. Fluorescence was quantified using a Bio Tek Synergy HTX microplate reader with filters at 530/590 nm. EdU fluorescence data were analyzed through ANOVA followed by post-hoc Tukey test.

**RNA Isolation and RNA Sequencing**

HUVECs were seeded with 250,000 cells at 90% confluency in a 6-well plate in triplicate for each treatment. After 12 hours the cells media was replaced with 1 mL of the appropriate treatment DMSO control or 50 μg/mL PFOS. The cells were exposed for 48 hours. After 48 hours the cells had the treatments removed and were exposed to 2-Mercaptoethanol, lysis buffer and were scraped with a cell scraper and then pipetted into a Qia shedder. The solution then followed the RNeasy mini kit from Qiagen. The quantity and quality of RNA was determined using a Thermoficher Qubit fluorometer. The isolated RNA was sent to the University of New Hampshire’s Hubbard Center for Genome Studies for library preparation and RNA sequencing. library preparation followed the KAPA mRNA HyperPrep kit using KAPA dual unique index along with Illumina Truseq adaptors. RNA sequencing was done using the Illuminia NovaSeq.

**Bioinformatics analysis**

All RNA-SEQ analysis was carried out on the Galaxy genomics platform (usegalaxy.org). Read quality was assessed using FastQC and reads less than 20 bp in length were filtered out. Reads were mapped to the reference human genome (hg38) using the HISAT program, counted using the featureCounts program, and subjected to further quality control checks using MultiQC. Counts from each sequencing file were joined and genes in the count matrix were annotated using the annotatemyIDs tool. Differential expression was compared using the limma-voom tool and lowly expressed genes were removed (minimum CPM = 0.5). Contrasts measured in limma-voom were Control versus PFAS and contrast testing was set to a minimum log _2_ fold-change of 0.58 and a *p* -value adjusted threshold of 0.01, significance was tested relative to a fold-change threshold. Gene ontology testing was performed using goseq and ENSEMBL gene set enrichment analysis was conducted with EGSEA and FGSEA.
